# Green synthesis, characterization and catalytic activity of natural bentonite-supported copper nanoparticles for the solvent-free synthesis of 1-substituted 1*H*-1,2,3,4-tetrazoles and reduction of 4-nitrophenol

**DOI:** 10.3762/bjnano.6.236

**Published:** 2015-12-03

**Authors:** Akbar Rostami-Vartooni, Mohammad Alizadeh, Mojtaba Bagherzadeh

**Affiliations:** 1 Department of Chemistry, Faculty of Science, University of Qom, P.O. Box 37185-359, Qom, Iran; 2 Material Research School, NSTRI, 81465-1589, Isfahan, Iran

**Keywords:** heterogeneous catalyst, modified bentonite, nanocomposite, 1**-**substituted 1*H***-**1,2,3,4**-**tetrazoles

## Abstract

In this study, Cu nanoparticles were immobilized on the surface of natural bentonite using *Thymus vulgaris* extract as a reducing and stabilizing agent. The natural bentonite-supported copper nanoparticles (Cu NPs/bentonite) were characterized by FTIR spectroscopy, X-ray diffraction (XRD), X-ray fluorescence (XRF), field emission scanning electron microscopy (FE-SEM), energy dispersive X-ray spectroscopy (EDS), transmission electron microscopy (TEM), selected area electron diffraction (SAED) and Brunauer–Emmett–Teller (BET) analysis. Afterward, the catalytic performance of the prepared catalyst was investigated for the solvent-free synthesis of 1**-**substituted 1*H***-**1,2,3,4**-**tetrazoles and reduction of 4-nitrophenol (4-NP) in water. It was found that the Cu NPs/bentonite is a highly active and recyclable catalyst for related reactions.

## Introduction

The development of new methodologies for the preparation of heterogeneous catalysts is of great interest in organic synthesis [1]. Metal nanoparticles immobilized on supports such as carbon, zeolites, clay, metal oxides, graphene, etc., have been successfully applied as heterogeneous catalysts due to their interesting structures and properties [2–6]. The extremely small scale of nanoparticles (NPs) is the main factor leading to their surprising reactivity as compared to their corresponding bulk metals [7]. However, most of these supports suffer from inefficiency to achieve highly distributed and stable metal NPs.

In recent decades, the use of natural bentonites has been studied due to their high specific surface area, low cost, ordered structure, thermal stability, high safety, high exchange capacity and intercalation abilities [8]. Smectites are major clay minerals in bentonite with an aluminum octahedral sheet sandwiched between two silica tetrahedral sheets [9]. These layered materials are very promising supports for the design and preparation of green catalysts [10].

Tetrazoles are among the heterocycles most applied in medicine and industry due to their structural potential such as their usage as an isosteric substituent for carboxylic acids [11], analytical reagents and biological applications [12–13]. Therefore, nowadays attention and progress in synthesis of these heterocycles play an essential role in organic, medicinal and synthetic chemistry [14–16]. During recent research on tetrazoles, 1**-**substituted 1*H***-**1,2,3,4**-**tetrazoles was found to be a special category due to their biological activity [16].

The plant biosynthesis of nanoparticles immobilized on natural supports is a subject of new research as little has been published on this topic [17–18]. Therefore, the use of plants as a natural and biological source for biosynthesis of nanoparticles should be explored.

**Scheme 1:**
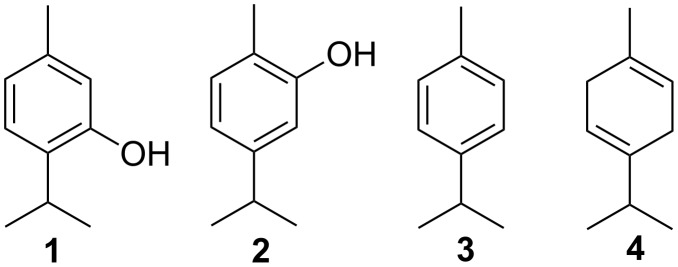
The major components of *Thymus vulgaris*. Thymol (**1**), carvacrol (**2**), *p*-cymene (**3**) and γ-terpinene (**4**).

Common thyme with the scientific name *Thymus vulgaris* is one of the plants indigenous to Iran which is valued for its antiseptic and antioxidant properties [19]. Thymol (**1**), carvacrol (**2**), *p*-cymene (**3**) and γ-terpinene (**4**) are the major components found in *Thymus vulgaris* (Scheme 1) [19].

As a continuation of our research on heterogeneous catalysts [20–21], we report a new protocol for the preparation of Cu NPs/bentonite by *Thymus vulgaris* extract and catalytic applications as a novel heterogeneous catalyst for the synthesis of 1**-**substituted 1*H***-**1,2,3,4**-**tetrazoles and reduction of 4-nitrophenol (4-NP). It was found that Cu NPs/bentonite is a highly active and recyclable catalyst for related reactions. The obtained results will be presented and described here.

## Experimental

### Instruments and reagents

All reagents were purchased from the Merck and Sigma-Aldrich and used without further purification. The bentonite and *Thymus vulgaris* plant used in this paper were collected from the Vartoon region (Isfahan, Iran). The IR spectra were recorded on a JASCO, FT/IR-6300 instrument in KBr pellets. The NMR spectra were obtained on a Brucker Avance 90 MHz spectrometer, using tetramethylsilane (TMS) as an internal standard. The melting points were taken in open capillary tubes with a Büchi 510 melting point apparatus and were uncorrected. Thin-layer chromatography (TLC) was performed on silica gel polygram SIL G/UV 254 plates. XRD analysis was performed on a Philips powder diffractometer type PW 1373 goniometer, which was equipped with a graphite monochromator crystal. The XRF analysis of the catalyst was performed with a Bruker S4 instrument. The morphology and particle dispersion was investigated by FE-SEM (Cam scan MV2300). The chemical composition of the modified bentonite was measured by EDX performed in a SEM. TEM images were obtained using a Philips-EM-2085 transmission electron microscope with an accelerating voltage of 100 kV. Nitrogen adsorption isotherms were performed on a volumetric gas adsorption apparatus (BEL Japan, Belsorp-max). The pore distributions and pore volumes were calculated using the adsorption branch of the N_2_ isotherms based on the Barrett–Joyner–Halenda (BJH) model. The specific surface area was calculated from the BET equation.

### Preparation of *Thymus vulgaris* extract

5 g of a dried powder of *Thymus vulgaris* leaves and pedicles was extracted by boiling in 30 mL double distilled water for 15 min and the aqueous extract was centrifuged at 7000 rpm to obtain the supernatant as an extract. This solution of the extract was used for the synthesis of Cu NPs/bentonite*.*

### Preparation of Cu NPs/bentonite

For green synthesis of Cu NPs/bentonite composite, 10 g natural bentonite was dispersed in 100 mL of 0.2 M CuSO_4_·5H_2_O under continuous stirring. After separation of quartz and feldspar precipitated in container, the above extract was added. This dispersion was stirred at 80 °C for 4 h. The prepared Cu NPs/bentonite was separated by filtration, washed several times with deionized water and absolute ethanol and dried at 100 °C for 2 h.

### General experimental procedure for the synthesis of 1-substituted 1*H*-1,2,3,4-tetrazoles

A mixture of amine (2 mmol), NaN_3_ (2 mmol), triethyl orthoformate (2.4 mmol) and Cu NPs/bentonite (0.05 g) was heated up to 120 °C for 3 h and stirred. After completion of the reaction (monitored by TLC), the reaction mixture was cooled to room temperature and diluted with cold water (5 mL) and extracted with ethyl acetate (3 × 10 mL). The catalyst was filtered and washed with water and ethanol. The combined organic layers were washed with brine and dried over the anhydrous MgSO_4_ and concentrated and crystallized with EtOAc-hexane to give different tetrazoles. All compounds were known and were characterized by spectral analysis or melting points [14].

### Catalytic reduction of 4-NP

Typically, 25 mL of 4-NP aqueous solution (2.5 mM) was mixed with 15 mg of the Cu NPs/bentonite in a beaker, stirring constantly for 2 min. Next, 25 mL of freshly prepared aqueous NaBH_4_ (0.25 M) was added and the mixture was allowed to stir at room temperature until the deep yellow solution became colorless. 1.0 mL of the solution was extracted and diluted to 25 mL for further UV–vis absorption analysis at certain intervals. The concentration of 4-NP was determined spectrophotometrically at a wavelength of 400 nm.

## Results and Discussion

### Preparation and characterization of Cu NPs/bentonite

The Cu NPs/bentonite composite was prepared by a simple and inexpensive method involving the immobilization of Cu NPs on bentonite using an aqueous extract of *Thymus vulgaris* without the usage of any special capping agents or surfactant template. The plant not only functioned as a reductant, but also served as a stabilizer for the formation of Cu NPs on the surface of natural bentonite. The obtained Cu NPs/bentonite was fully characterized by XRF, FTIR, XRD, FE-SEM, EDX, TEM, SAED and BET analyses.

**Figure 1:**
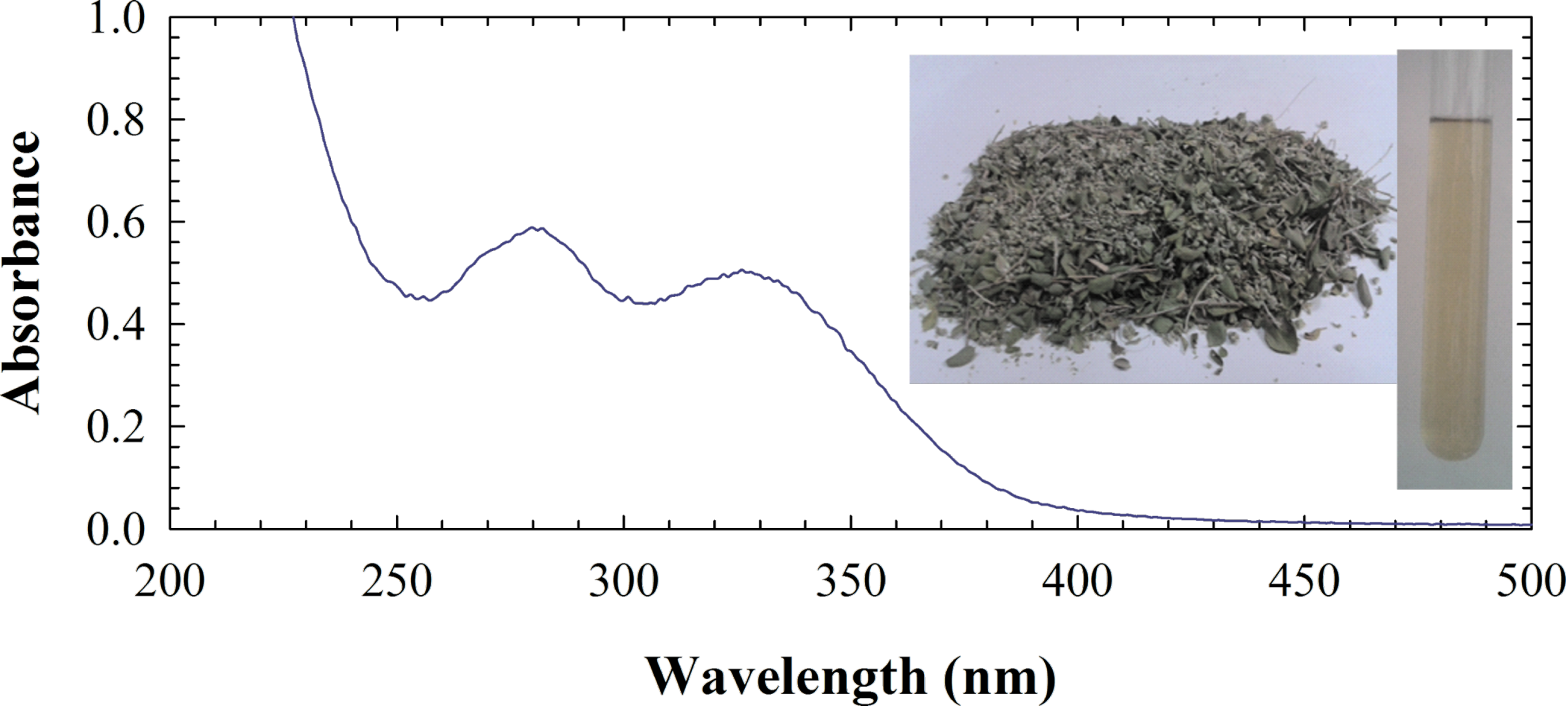
UV–vis spectrum of an aqueous extract of *Thymus vulgaris*.

As depicted in Figure 1, the UV–vis spectra of the plant showed π→π* transitions of aromatics in the extract. Also, given the presence of monoterpenes such as thymol and carvacrol, these transitions are probably related to the mentioned compounds involved in the reduction process and formation of copper nanoparticles deposited on bentonite surface through π-electron interactions [17,21]. Hence, the extract of *Thymus vulgaris* acts as the reductant as well as the stabilizer.

The XRF results in Table 1 show the variations in the chemical composition of bentonite upon successful modification with Cu (4.3 wt %). The presence of Cu and the absence of Na_2_O in these results as compared with the XRF results of natural bentonite [22] were due to ion exchange of Na^+^ ions from the bentonite with Cu^2+^ ions, followed by reduction under *Thymus vulgaris* extract. On the other hand, the reduction and ion exchange processes are simultaneous.

**Table 1:** Typical approximate compositional analysis of Cu NPs/bentonite.

Constituent	Typical (%)

SiO_2_	62.71
Al_2_O_3_	12.3
K_2_O	0.47
CaO	2.06
MgO	1.35
Fe_2_O_3_	1.06
TiO_2_	0.07
Cu	4.27
LOI^a^	12.55

^a^LOI: loss on ignition (1000 °C, 2 h).

**Figure 2:**
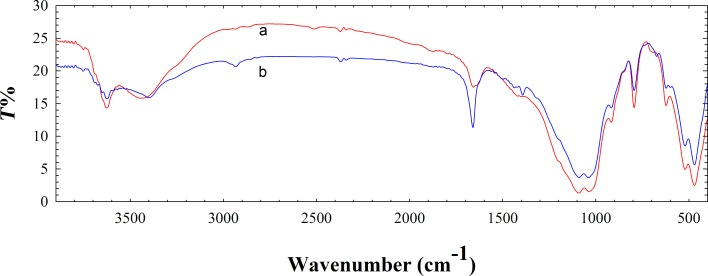
FTIR spectra of (a) natural bentonite and the (b) Cu NPs/bentonite composite.

FTIR spectra of the natural (a) and modified (b) bentonite are presented in Figure 2. As can be seen in Figure 2, four typical groups of IR bands can be clearly distinguished in both spectra. These have been previously reported and assigned to the following major vibrations: below 400 cm^−1^, lattice vibrations occur, while pseudo-lattice vibrations are observed at about 500–700 cm^−1^; internal vibrations of Si–O(Si) and Si–O(Al) in tetrahedral or alumino- and silico-oxygen bridges emerge in the range between 400 and 1200 cm^−1^, whereas the bands of adsorbed water and surface hydroxyls are evident at about 1630–1650 cm^−1^ and 3000–3800 cm^−1^, respectively [23–27]. The structural modifications of the tetrahedral and octahedral sheets due to the adsorbed Cu(II) cations and formation of Cu NPs on bentonite influenced the fundamental vibrations of the Si–O and H_2_O groups (Figure 2b). From the FTIR spectrum of the modified bentonite sample (Figure 2b), it is notable that the broad band of water near 3430 cm^−1^ has shifted to higher frequencies in comparison to the natural bentonite (Figure 2a). Also, a new peak at 1385 cm^−1^ is observed for modified bentonite, the origin of which was interpreted in terms of substitution of the naturally present alkaline metals with Cu(II) ions as a result of the modification [28]. The observed results confirmed that the structural changes were acquired during the modification of bentonite. Further proof was obtained by the XRD and EDS results.

**Figure 3:**
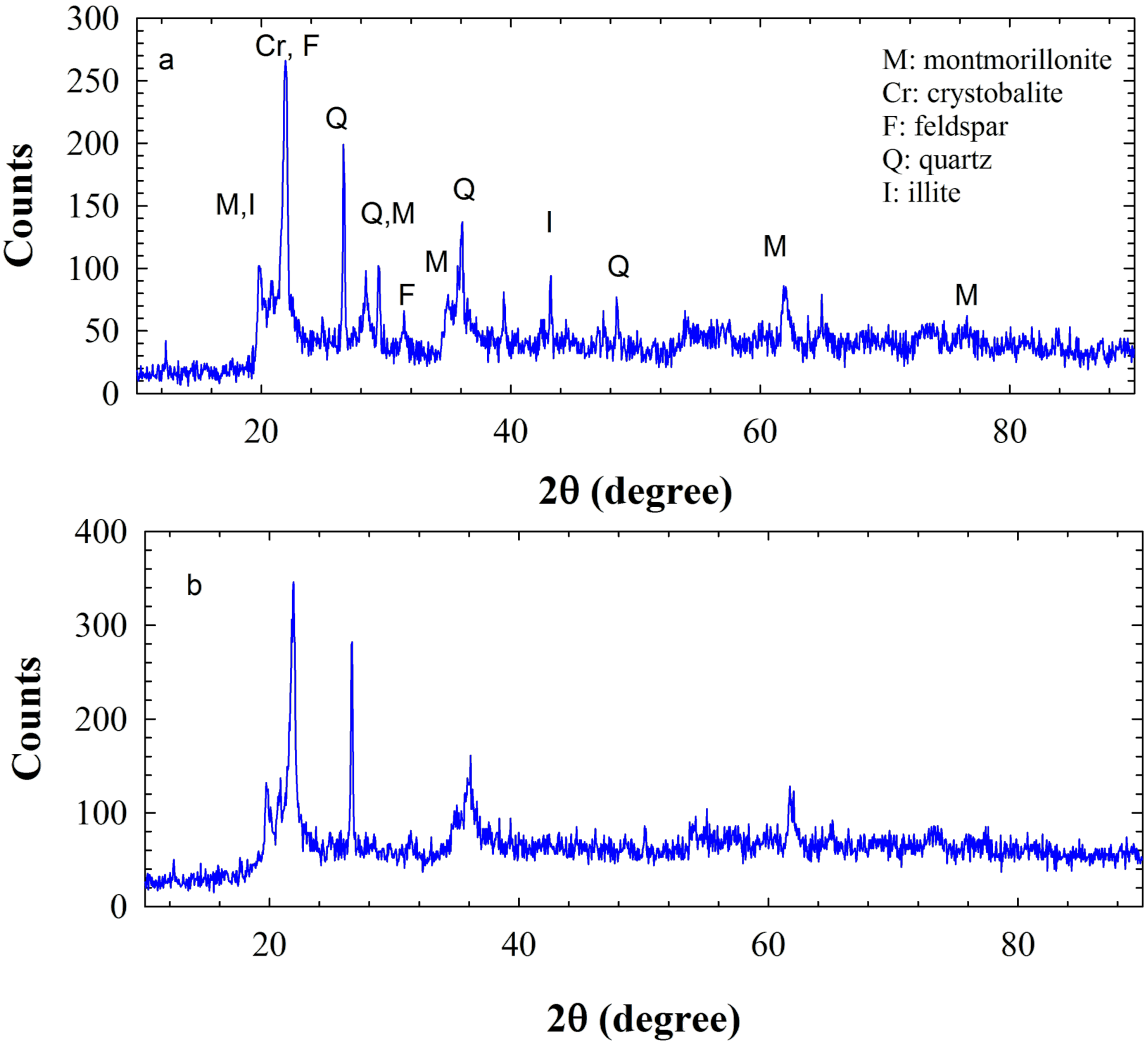
The XRD patterns of natural bentonite (a) and Cu NPs/bentonite (b).

The XRD patterns of the raw bentonite (Figure 3a) and modified bentonite (Figure 3b) are shown in Figure 3. The XRD pattern in Figure 3a reveals that the principal constituents of the employed natural clay are montmorillonite (M) and quartz (Q), where the characteristic peaks located at 2θ = 19.84, 34.80, and 61.84° were indexed to (020), (130), and (060) planes of montmorillonite, respectively, and 2θ = 26.81, 36.25, and 48.84° were indexed to (101), (110), and (201) planes of quartz, respectively. The other peaks are impurities corresponding to crystobalite, feldspar and illite [25]. It is expected that after modification of bentonite with Cu, a number of new peaks appear at 2θ = 40–55° [29]. However, as can be seen in the XRD pattern of Figure 3b, no specific peaks were obtained for the Cu NPs. This might be due to the low percentage or high dispersion of Cu NPs in the bentonite matrix. However, the existence of Cu NPs can be confirmed by the EDS technique.

**Figure 4:**
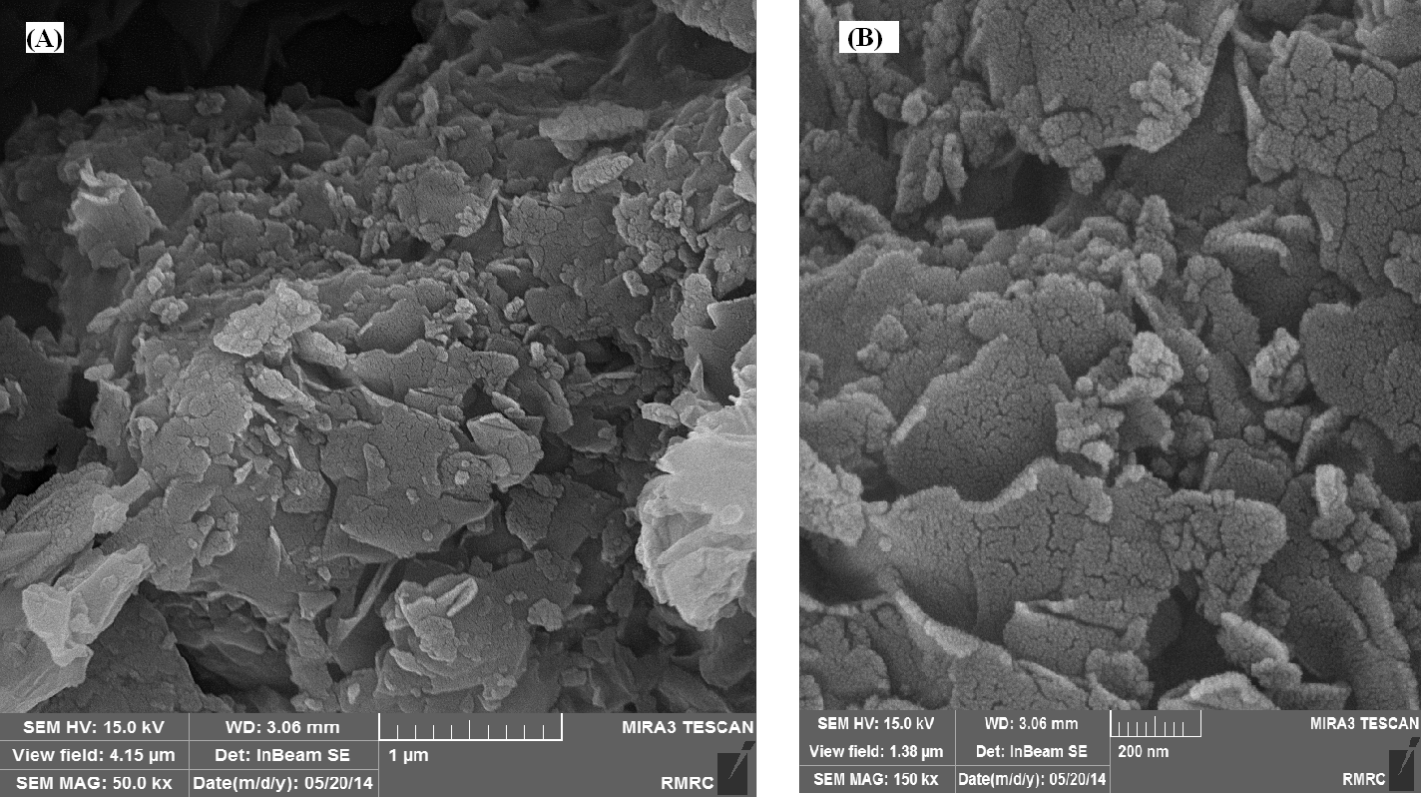
Typical FE-SEM images of Cu NPs/bentonite. Scale bar: (a) 1 µm and (b) 200 nm.

**Figure 5:**
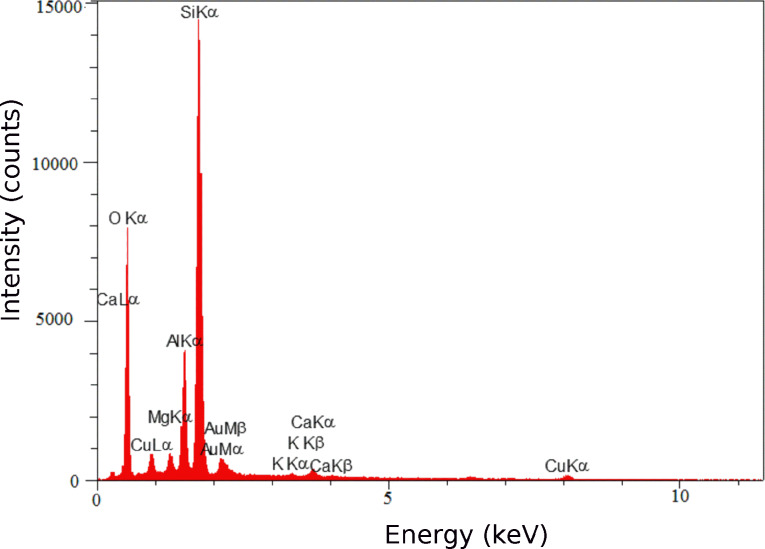
EDS spectrum of Cu NPs/bentonite.

Figure 4 shows the FE-SEM of Cu NPs/bentonite. The sheet-like structure of montmorillonite can be seen in SEM images. These images show that the adsorption of Cu NPs can occur on both the external surface and interlayer spaces. However, in the EDS spectrum (Figure 5), peaks related to Cu (6.07 % w/w), Si, Al, Mg and O were observed.

**Figure 6:**
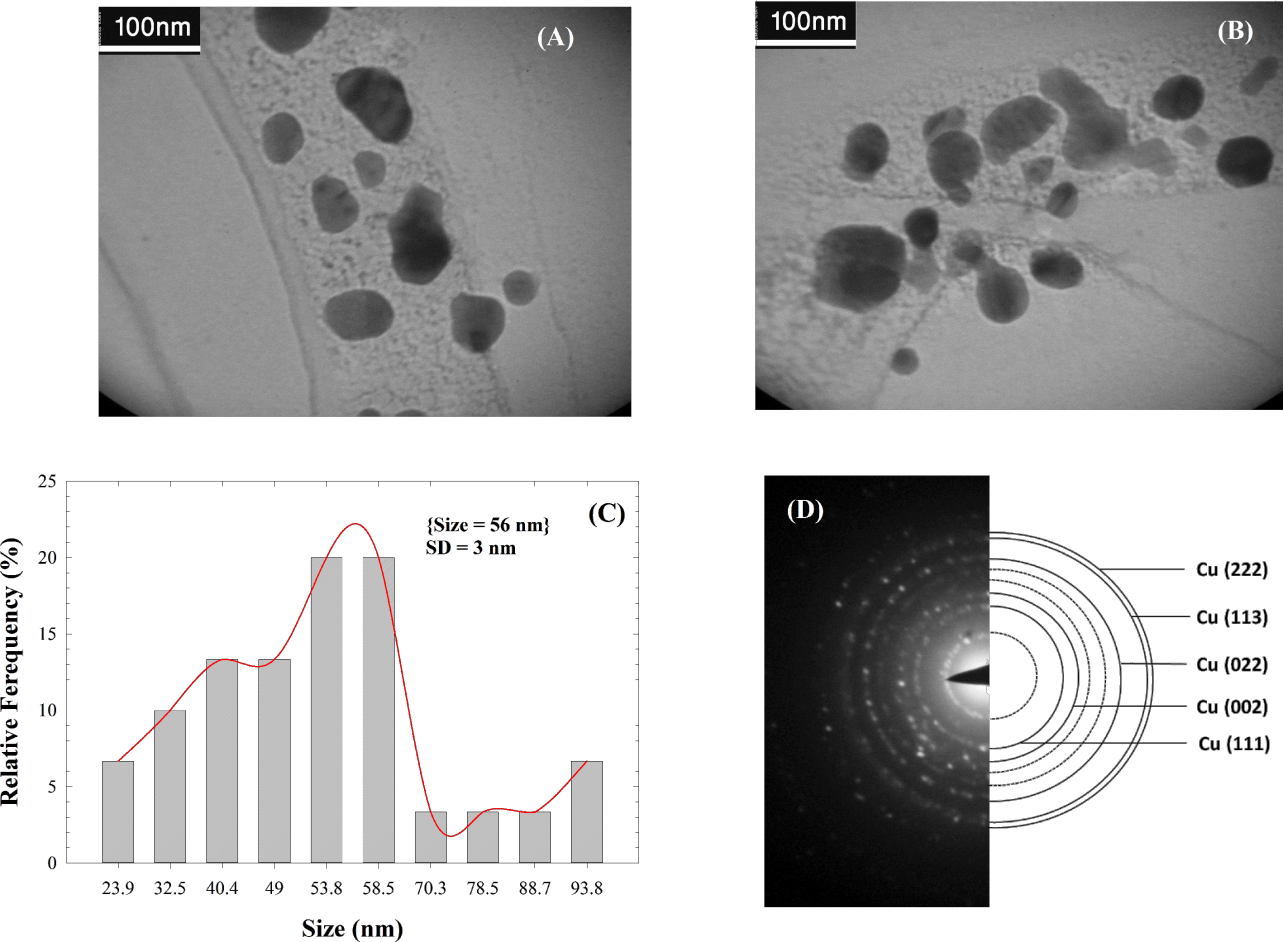
Typical TEM images of Cu NPs/bentonite (a,b), the histogram of the particle size distribution of Cu nanoparticles on the bentonite surface (c) and corresponding SAED pattern (d).

The size of the as-prepared Cu NPs/bentonite was further examined by TEM. The histogram of the particle size distribution of Cu nanoparticles on the surface of bentonite is given in Figure 6a–c. The average size of the Cu NPs on bentonite was 56 nm. The particles exhibited spherical morphology with a low tendency for agglomeration. Figure 6d (SAED) shows the measured selected area electron diffraction pattern of as-prepared Cu NPs/bentonite. This result indicates that the nanoparticles are crystalline and mainly composed of fcc Cu. The SAED patterns of the Cu NPs/bentonite sample are used to characterize the planes, interplanar spacing, nanostructure and zone axis. These results verified the successful synthesis of Cu NPs on bentonite. In the presence of the *Thymus vulgaris* extract as a reducing and stabilizing agent, the Cu^2+^ ions convert to Cu NPs and immobilize on the surface of bentonite [6,20,30–33].

**Figure 7:**
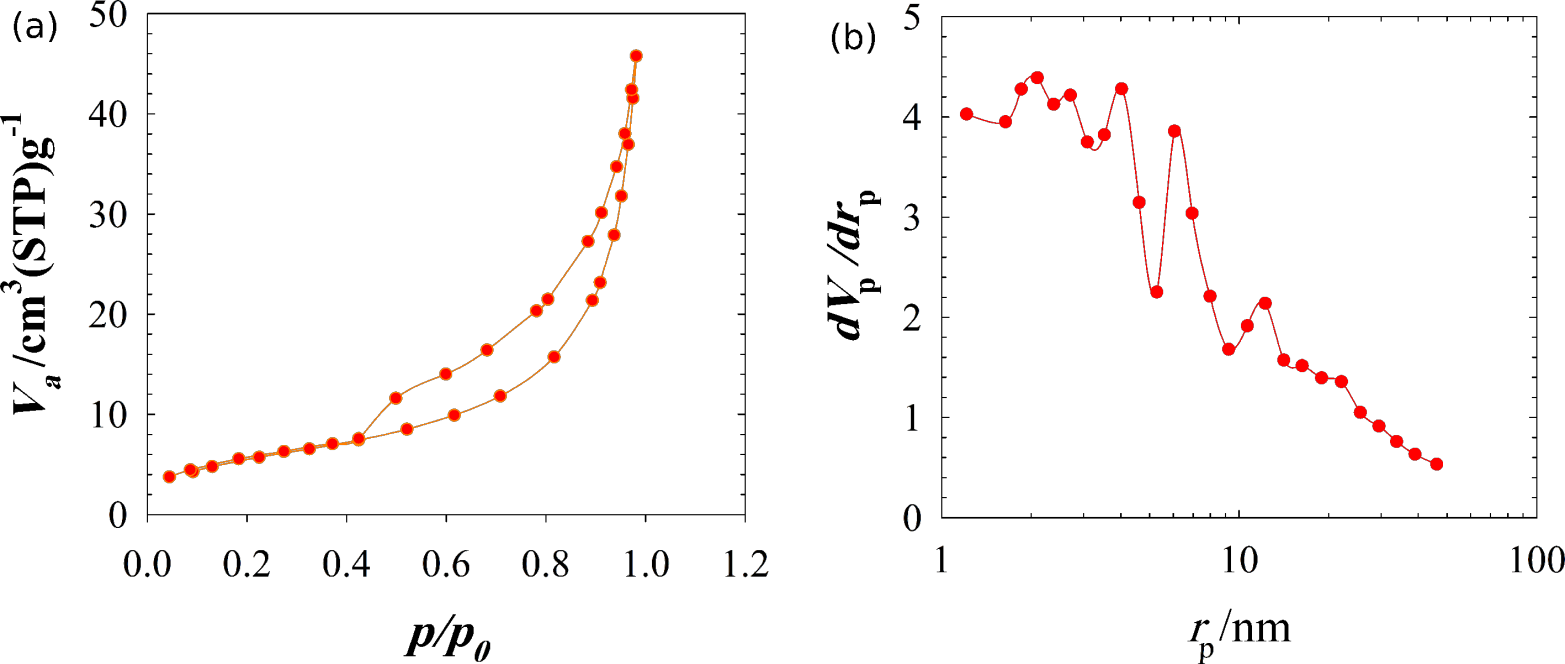
The N_2_ adsorption–desorption isotherm (a) and Barrett–Joyner–Halenda (BJH) pore size distribution plot of Cu NPs/bentonite (b).

The surface area measurements were performed on Cu NPs/bentonite. Figure 7 shows the N_2_ adsorption–desorption isotherm and BJH pore size distribution plot of Cu NPs/bentonite. The results indicate that the surface area, total pore volume and average pore diameter were 19.1 m^2^/g, 0.071 cm^3^/g and 14.79 nm, respectively. For similar Iranian bentonite [34], the surface area, total pore volume and average pore diameter were 31.8 m^2^/g, 0.093 cm^3^/g and 11.7 nm, respectively. The surface area and total pore volume of Cu NPs/bentonite decreased compared to the natural bentonite, whereas its pore size increased.

### Activity of Cu NPs/bentonite for the synthesis of 1-substituted 1*H*-1,2,3,4-tetrazoles

**Scheme 2:**
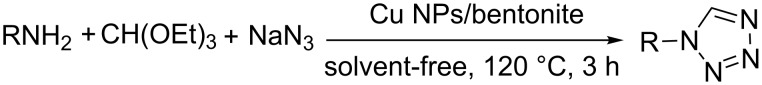
Synthesis of 1-phenyl-1*H*-1,2,3,4-tetrazole (R = phenyl).

For the further understanding of the role of Cu NPs/bentonite, a comprehensive study of the synthesis of 1**-**substituted 1*H***-**1,2,3,4**-**tetrazoles was carried out (Scheme 2).

Initial studies were performed in order to optimize the reaction conditions for the synthesis of 1-phenyl-1*H*-1,2,3,4-tetrazole. As proved by control experiments, no reaction occurs in the absence of Cu NPs/bentonite. The best result was obtained with the with 2.0:2.0:2.4 molar ratio of aniline/sodium azide/triethyl orthoformate, in the presence of Cu NPs/bentonite (0.05 g) under solvent-free conditions at 120 °C.

A series of primary aromatic amines were converted into the corresponding 1-substituted tetrazoles with sodium azide and triethyl orthoformate using Cu NPs/bentonite in high yields under thermal and solvent-free conditions (Table 2). The influence of various substituents in different *ortho*, *meta* or *para* positions on the type of products were examined. Amines containing both electron-releasing and electron-withdrawing groups underwent the conversion in good to excellent yield.

**Table 2:** Preparation of 1**-**substituted 1*H***-**1,2,3,4**-**tetrazoles in the presence of Cu NPs/bentonite by reaction between sodium azide, primary amines and triethyl orthoformate at 120 °C.^a^

Entry	Substrate	Product	Yield [%]^b^

1	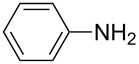	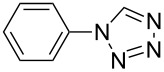	93
2	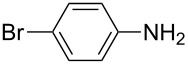	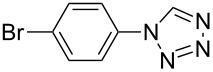	85
3	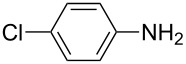	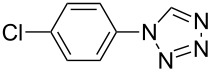	94
4	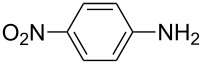	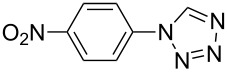	87
5	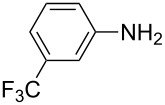	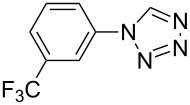	83
6	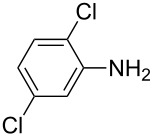	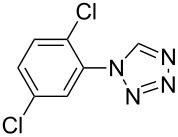	85
7	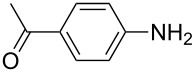	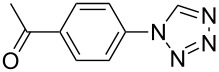	87
8	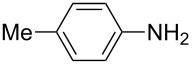	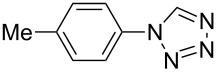	87
9	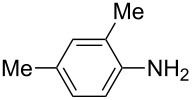	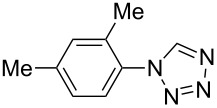	88
10	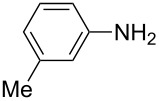	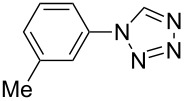	92
11	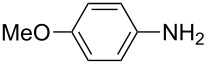	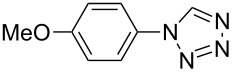	86
12	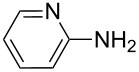	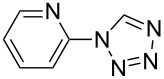	83

^a^Reaction conditions: Amine (2.0 mmol), NaN_3_ (2 mmol), triethyl orthoformate (2.4 mmol), Cu NPs/bentonite (0.05 g) at 120 °C, 3 h. ^b^Isolated yield.

**Scheme 3:**
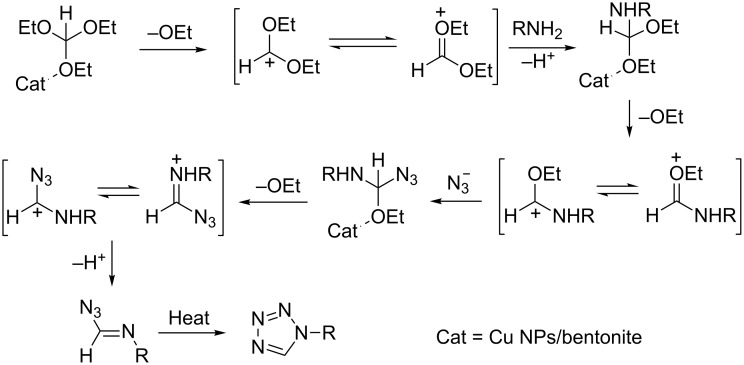
The proposed mechanism for the preparation of 1-phenyl-1*H*-1,2,3,4-tetrazole.

The Cu NPs/bentonite likely plays an important role in the preparation of 1-substituted 1*H*-1,2,3,4-tetrazole as a Lewis acid and the plausible mechanism is shown in Scheme 3 [14]. The breakdown of the C–OEt bond in triethyl orthoformate facilitates the elimination of EtOH and the final 1-substituted 1*H*-1,2,3,4-tetrazole is produced. The energy of these cleavages and formation of products are provided by the heat (Scheme 3).

**Figure 8:**
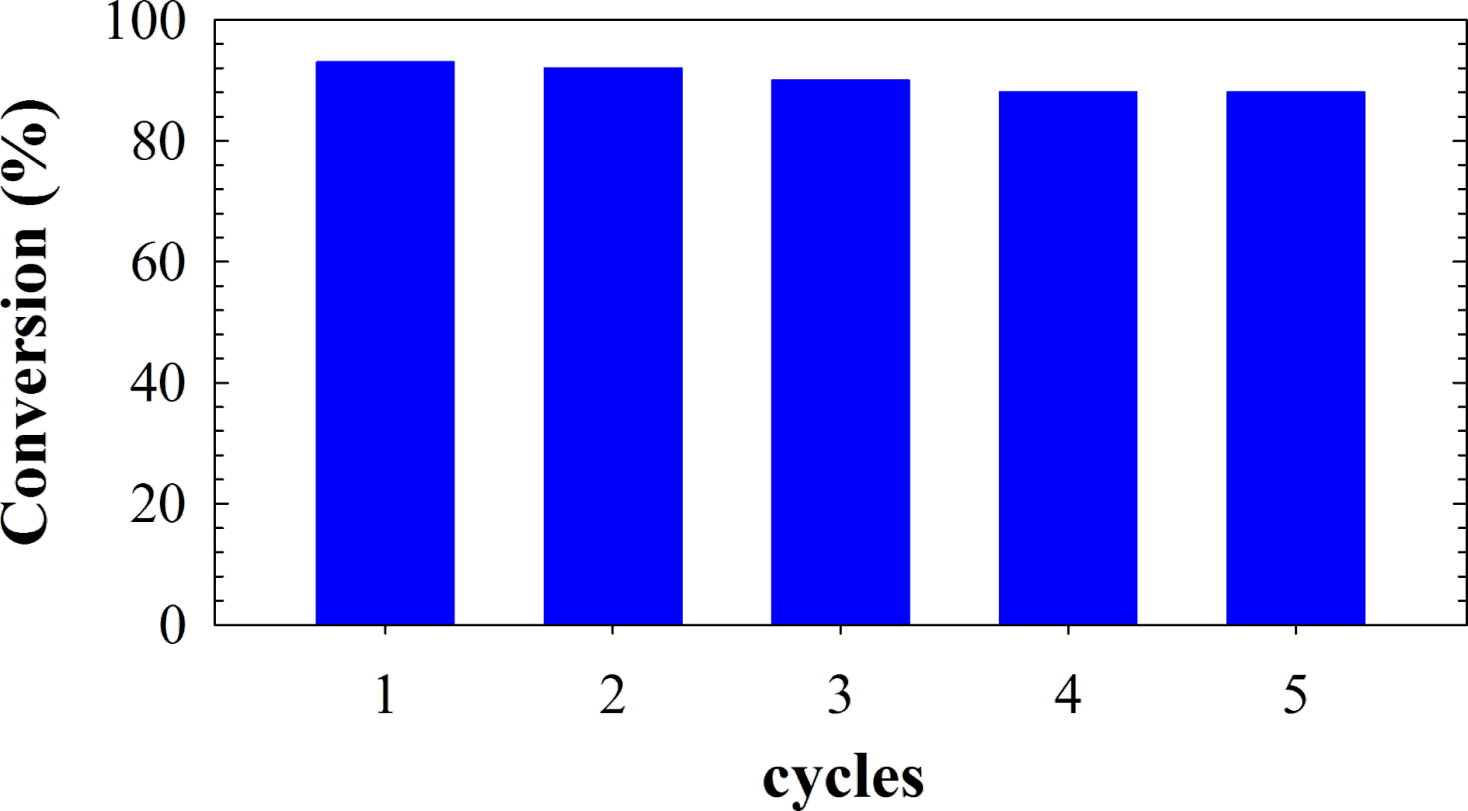
Conversion against number of catalytic cycles for the preparation 1-phenyl-1*H*-1,2,3,4-tetrazole with Cu NPs/bentonite for five successive cycles. Conditions: aniline (2 mmol), NaN_3_ (2 mmol), triethyl orthoformate (2.4 mmol), catalyst (0.05 g) at 120 °C, 3 h.

The recyclability of Cu NPs/bentonite in the preparation of 1-phenyl-1*H*-1,2,3,4-tetrazole was also investigated. As shown in Figure 8, no significant decrease in catalytic activity was observed for the recovered catalyst after four catalytic cycles. As compared with other literature works on the synthesis of 1-substituted 1*H*-1,2,3,4-tetrazole [35], the present work is comparable because it was carried out without solvent, at low reaction time, in the presence of a catalyst prepared from bentonite as an inexpensive and natural compound.

### Application of the Cu NPs/bentonite for the catalytic reduction of 4-NP

**Figure 9:**
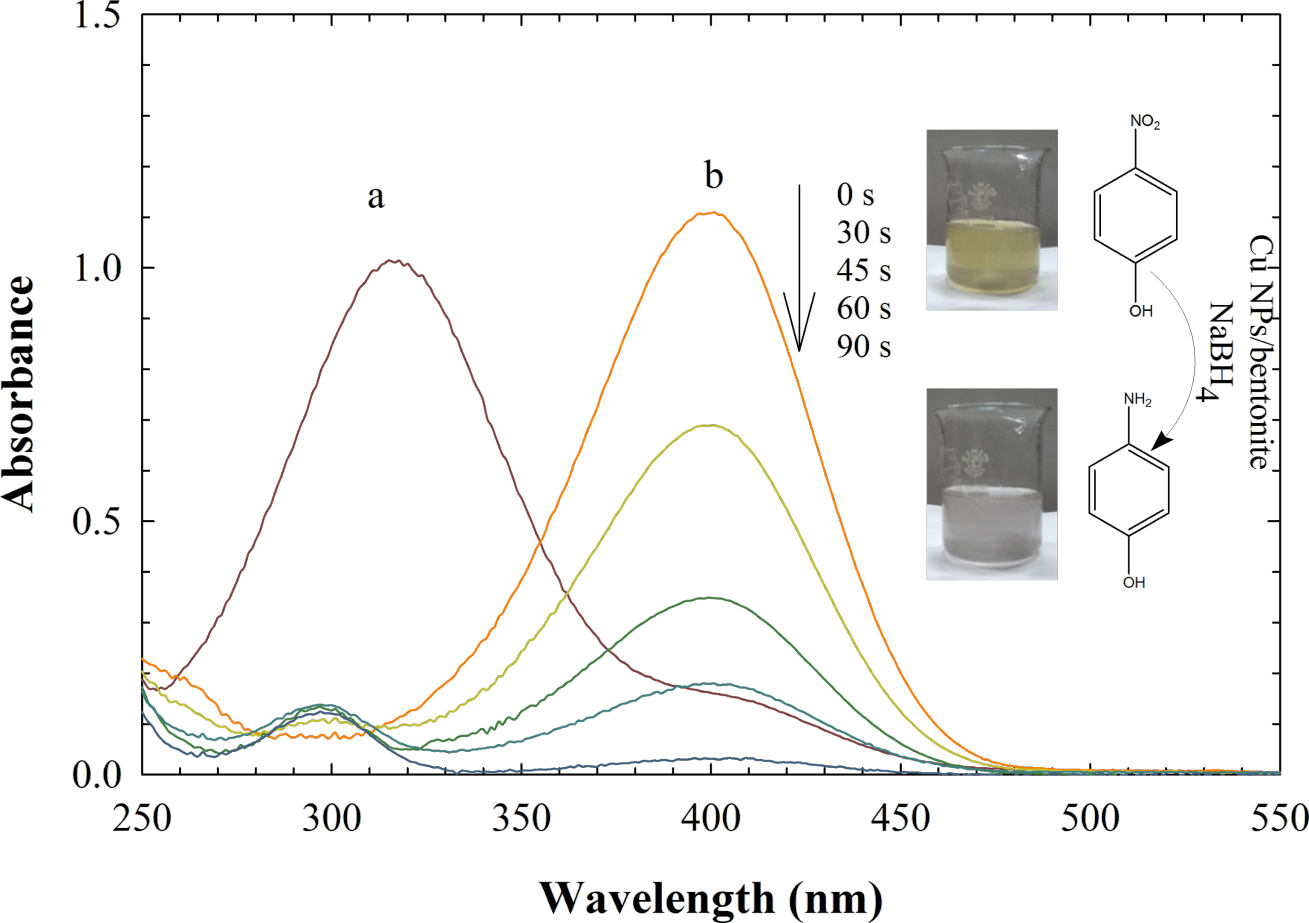
UV–vis absorption spectra of 4-NP (a) and 4-NP + NaBH_4_ (b) at several intervals. Conditions: [4-NP] = 2.5 × 10^−3^ M; [NaBH_4_] = 0.25 M; catalyst = 15 mg.

Another reaction chosen as a model reaction to evaluate the catalytic activity of the Cu NPs/bentonite was the catalytic reduction of 4-NP to 4-aminophenol in the presence of NaBH_4_. The concentration of 4-NP was monitored at given intervals by using UV–vis spectroscopy and the results are shown in Figure 9. The original absorption peak of 4-NP is centered at about 320 nm and shifts to 400 nm after addition of the NaBH_4_ solution. This is due to the formation of *p*-nitrophenolate ions under alkaline conditions with NaBH_4_ [36]. The absorption peak at 400 nm fully disappeared in the presence of 15 mg of catalyst after a 90 s induction period. In similar conditions, the study was carried out at lower concentrations of NaBH_4_. The reaction time was 420 and 515 s in the presence of 2.5 × 10^−2^ and 1.3 × 10^−2^ M NaBH_4_, respectively. In addition, a reference experiment of Cu NPs/bentonite with only 4-NP was carried out. After 12 h, the 4-NP could not be reduced to 4-AP by the catalyst in the absence of NaBH_4_. The catalyst could be recovered and reused several times without significant loss of catalytic activity. In the presence of natural bentonite (15 mg), no significant color change was observed in a similar reduction process within 3 h. The catalytic reduction process using Cu NPs/bentonite can be summarized as follows: diffusion and adsorption of both BH_4_^−^, as a strong nucleophile, and 4-NP onto the Cu surface; followed by electron transfer from the BH_4_^−^ to 4-NP; and finally, desorption of the generated 4-aminophenol from the surface of the catalyst [37–38].

**Figure 10:**
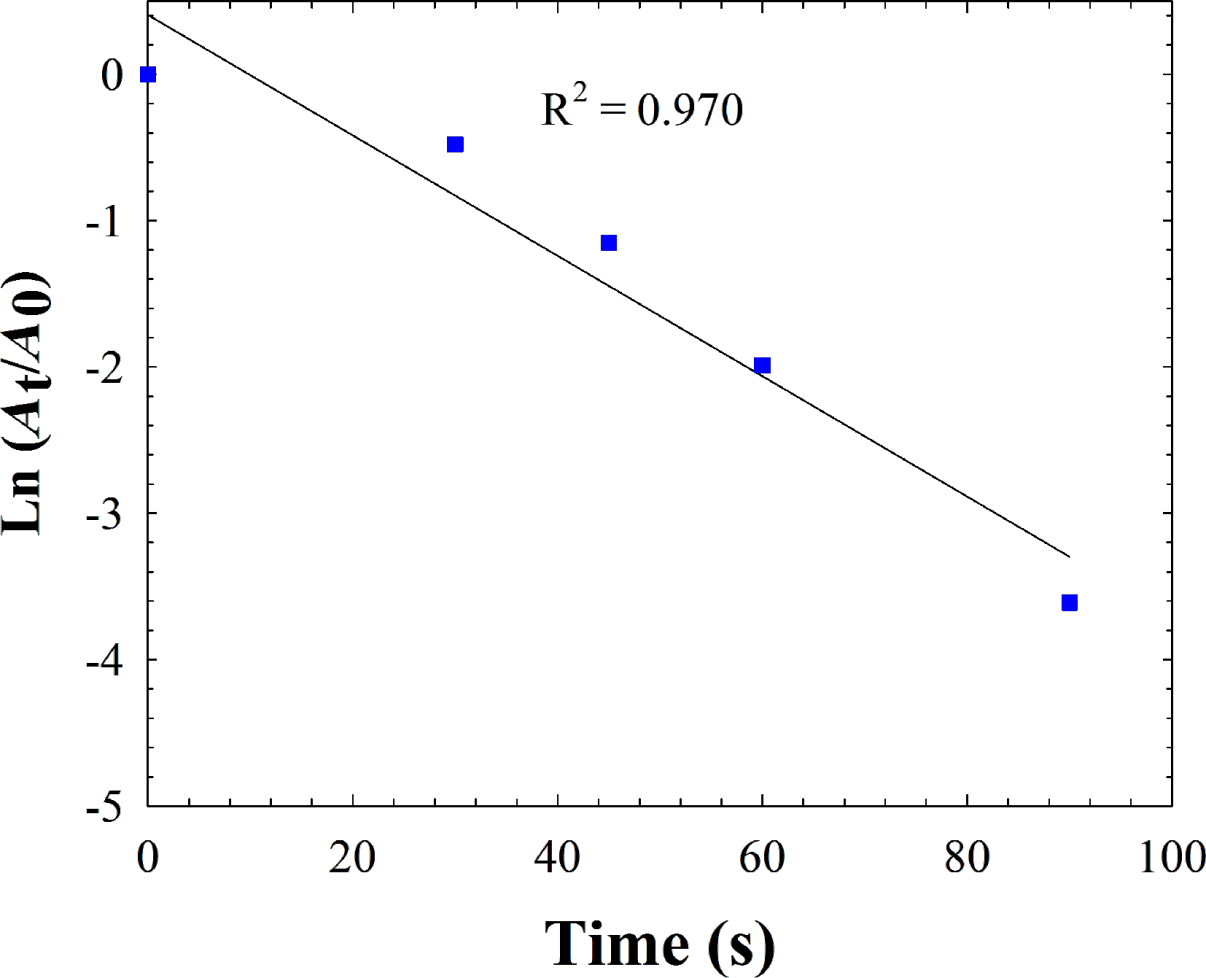
Plot of ln(*A*_t_/*A*_0_) vs irradiation time for 4-NP reduction. Conditions: [4-NP] = 2.5 × 10^−3^ M; [NaBH_4_] = 0.25 M; catalyst = 15 mg.

Since catalysis takes place on the Cu surface, Cu NPs/bentonite are much more reactive than the unmodified natural bentonite. Furthermore, it is found that the reduction of 4-NP over catalyst in the presence of a large excess of NaBH_4_ compared to 4-NP can be treated by a pseudo-first-order equation [18]: ln(*C*_t_/*C*_0_) = ln(*A*_t_/*A*_0_) = −*kt*, where *C*_t_ is the concentration of 4-NP at a reaction time *t*, *C*_0_ is the initial concentration of 4-NP, *A*_t_ is the absorbance at any time *t*, and *A*_0_ is the absorbance at time *t* = 0. From the linear relations of ln(*A*_t_/*A*_0_), shown in Figure 10, we found that the rate constant (*k*) for this reaction is 0.041 s^−1^, which is comparable to that previously reported [39–42]. The enhanced reactivity of the Cu NPs/bentonite is related to the dispersion of Cu NPs particles in the support, which provides more accessible reactive sites for reduction of 4-NP.

## Conclusion

In this study, copper nanoparticles supported on natural bentonite using a *Thymus vulgaris* extract as a reducing and stabilizing agent were prepared and characterized. This catalyst was found to be an efficient and recyclable heterogeneous catalyst for the synthesis of 1**-**substituted 1*H***-**1,2,3,4**-**tetrazoles and reduction of 4-NP under mild conditions. The Cu NPs/ bentonite composite remained stable under several reactions.
